# Safety and Effectiveness of Mavacamten Use in Hypertrophic Obstructive Cardiomyopathy: A Systematic Review and Meta-Analysis

**DOI:** 10.7759/cureus.70550

**Published:** 2024-09-30

**Authors:** Tuqa Y Alharbi, Hadel A Alnadawi, Ghadah M Almutairi, Fatimah Y Altheyab, Osama H Aldoweesh, Omar S Alfehaid, Abdulmalik A Alhaj, Abdulaziz M Alotaibi, Ali M Al Zweihary

**Affiliations:** 1 College of Medicine, Qassim University, Qassim, SAU

**Keywords:** cardiac myosin inhibitor, cardiac remodeling, h.o.c.m, l.v.o.t gradient, mavacamten

## Abstract

Hypertrophic obstructive cardiomyopathy (HOCM) is a complex genetic cardiac disease that causes left ventricular hypertrophy and obstruction of the outflow tract. Mavacamten, a novel cardiac myosin inhibitor, has emerged as a potentially beneficial therapeutic option. This meta-analysis aimed to determine whether mavacamten is effective and safe for use in patients with HOCM.

A systematic literature search was performed in PubMed and the Cochrane Central Register of Controlled Trials to identify randomized controlled trials (RCTs) that compared mavacamten to placebo in patients with HOCM. The primary objectives were changes in the gradients associated with the Valsalva maneuver and resting left ventricular outflow tract (LVOT). Alterations in the left atrial volume index (LAVI), left ventricular mass index (LVMI), and NT-proBNP level were secondary outcomes. Safety outcomes were also evaluated. Random effects models were used in the meta-analysis.

Two RCTs comprising 332 patients were included. Mavacamten significantly reduced the Valsalva LVOT gradient (mean difference (MD) = -54.94 mmHg; 95% CI: -70.32, -39.56; P = 0.13) and resting LVOT gradient (MD = -42.44 mmHg; 95% CI: -67.52, -17.36; P<0.001) compared to placebo. Significant improvements were also observed in LAVI (MD = -7.18 mL/m²; 95% CI: -11.00, -3.37; P = 0.24) and NT-proBNP levels (RR = 0.58; 95% CI: 0.39, 0.84; P<0.001). LVMI showed a trend toward reduction (MD = -19.15 g/m²; 95% CI: -41.98, 3.69; P<0.001). Mavacamten demonstrated a favorable safety profile with few reported adverse events.

This meta-analysis aimed to demonstrate the efficacy and short-term safety of mavacamten in patients with HOCM. Considerable improvement was observed in the LVOT gradients, cardiac remodeling measures, and indicators of cardiac stress when mavacamten was administered. Based on this data, mavacamten appears to offer the potential for a paradigm shift in the management of HOCM. However, studies conducted over an extended period are required to validate its long-term effectiveness and safety profile.

## Introduction and background

Hypertrophic obstructive cardiomyopathy (HOCM) is a genetically diverse heart condition characterized by abnormal myocardial hypertrophy, especially of the left ventricle, which impairs diastolic and systolic function and causes dynamic left ventricular outflow tract (LVOT) obstruction [1-2]. This disorder, which is primarily inherited in an autosomal dominant pattern, can present clinically in a variety of ways, from asymptomatic individuals to those with severe heart failure, arrhythmias, or sudden cardiac death [2-3]. HOCM is a dynamic LVOT obstruction with systolic anterior motion of the mitral valve, which can be observed on echocardiography and MRI [3-4]. Since mutations in multiple genes encoding sarcomeric proteins are known to be involved in the etiology of the disease, genetic testing is also essential for the diagnosis and treatment of HOCM [5]. Prior to the introduction of myosin inhibitors, there were limited treatment options. Only anecdotal use of beta-blockers, nondihydropyridine calcium channel blockers, and septal reduction therapy (either alcohol septal or surgical) were available. No randomized trial has been conducted on the use of beta-blockers or calcium channel blockers, emphasizing the need for innovative treatment solutions [6].

Mavacamten, previously known as MYK-461, is a recently discovered, innovative small-molecule modulator of cardiac myosin that addresses the underlying sarcomere hypercontractility in hypertrophic cardiomyopathy [7]. Mavacamten reduces the number of myosin heads that can enter the on-actin (power-generating) state, thereby reducing the chance of cross-bridge formation in HCM, and shifts the overall myosin population to the energy-saving 'off state' [8]. Recent research has shown that mavacamten not only improves hemodynamic parameters but also enhances quality of life and exercise tolerance in patients with HOCM [9]. Furthermore, mavacamten has been demonstrated to reduce the need for invasive procedures such as septal reduction therapy, presenting a safer alternative for symptom management [9-10].

This meta-analysis aimed to determine the effectiveness of mavacamten in improving the condition of patients with HOCM and to assess its efficacy in managing the symptoms and progression of the disease. We evaluated the safety profile of mavacamten, focusing on potential adverse effects and its overall tolerability among patients with HOCM. Additionally, to conduct a comparative analysis between mavacamten and other drugs commonly used for treating HOCM, we examined outcomes and effectiveness to provide valuable insights into the best treatment options for these patients.

## Review

Methods

Search Strategy and Study Selection

A comprehensive literature search was performed using PubMed and the Cochrane Central Register of Controlled Trials (CENTRAL). The search was conducted using the following Medical Subject Headings (MeSH) phrases and keywords: "Mavacamten," "cardiac myosin inhibitor," "hypertrophic obstructive cardiomyopathy," "left ventricular outflow tract obstruction," and "randomized controlled trials." Two reviewers performed the search independently to ensure thoroughness and minimize the possibility of bias.

A study was included if it met the following criteria: 1) RCTs involving people with HOCM; 2) mavacamten treatment compared to placebo or standard care; 3) published in English; and 4) outcomes relevant to the clinical question, such as measures of cardiac function and drug safety. Studies were excluded based on specified criteria, such as lack of RCTs, duplicate publications, studies with a high risk of bias, or studies with insufficient statistical significance. A PRISMA flow diagram was developed to describe the research selection process.

Data extraction and quality assessment 

Two reviewers used a standardized form to collect data from the studies. The retrieved data included study characteristics, such as the author, year of publication, country, research method, sample size, and follow-up duration.

Patient characteristics included age, sex, race, body weight, BMI, underlying diseases (such as hypertension or dyslipidemia), current medications, and treatment duration. The treatment specifics, such as mavacamten dosage and treatment period, were also collected.

This study examined various outcome indicators, including changes in the Valsalva LVOT gradient, resting LVOT gradient, left atrial volume index (LAVI), left ventricular mass index (LVMI), NT-proBNP level, and echocardiographic parameters. Safety assessment results, including adverse events, were also documented.

The Cochrane risk-of-bias tool for randomized trials was used to assess the methodological reliability and potential bias of the publications included in the review. This tool examined seven areas: random sequence generation, allocation concealment, participant and staff blinding, blinding of outcome assessment, incomplete outcome data, selective reporting, and other biases. Each domain was classified as low, high, or unclear risk of bias. Any disagreements between the reviewers were resolved through discussion or consultation with a third reviewer. The Jadad Scale and the CONSORT Checklist were also used for quality assessment.

Statistical analysis

A meta-analysis was performed to account for the heterogeneity among the studies, using a random-effects model. The mean differences (MD) for continuous outcomes, such as the Valsalva LVOT gradient, resting LVOT gradient, LAVI, and LVMI, were computed. CIs at the 95% confidence level were calculated. The conventional inverse variance method was used to determine the geometric mean ratio of NT-proBNP levels.

The I² statistic was used to calculate the level of heterogeneity, with values less than 25% indicating low heterogeneity, values between 25% and 75% indicating moderate heterogeneity, and values greater than 75% indicating high heterogeneity. Statistical significance was set at p < 0.05. Forest plots were developed for each result to visually display the data, including individual and pooled effect sizes and 95% CIs.

Funnel plots were used to improve the measurement of publication bias, and these were visually examined to identify any potential bias. Due to the small number of papers included in the analysis, no rigorous statistical tests for publication bias, such as Egger's test, were performed. A sensitivity analysis was conducted to assess the reliability of the results, but because of the small number of publications, neither a subgroup analysis nor these studies were conducted.

Statistical analysis for this study was conducted using Review Manager (RevMan) version 5.4, as recommended by The Cochrane Collaboration in 2020. The meta-analysis followed the Preferred Reporting Items for Systematic Reviews and Meta-Analyses (PRISMA) standards to ensure proper methodology.

Results

Search Results

**Figure 1:**
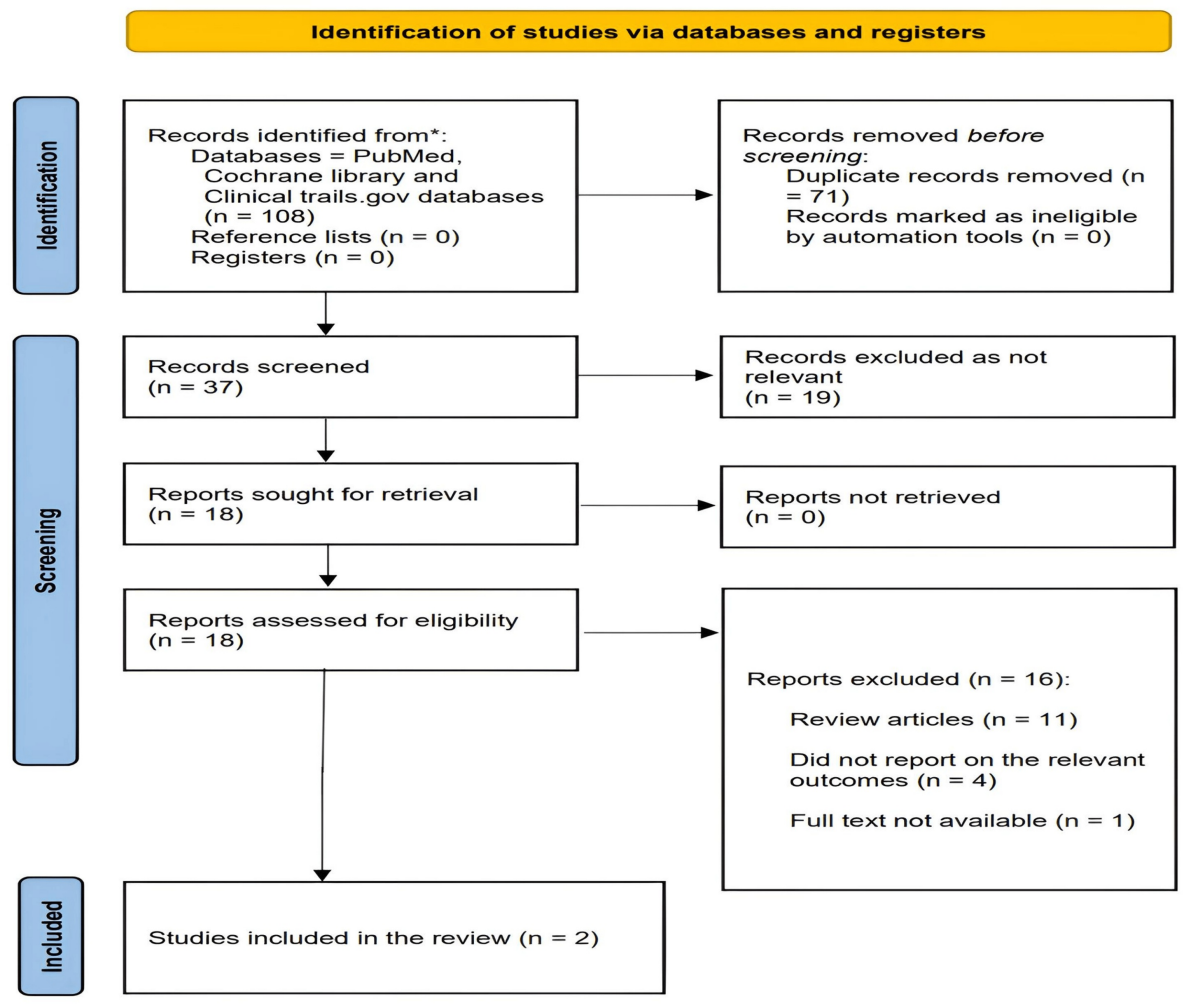
PRISMA flowchart of included studies. PRISMA: Preferred Reporting Items for Systematic Reviews and Meta-Analyses.

A flowchart illustrating the study selection process is shown in Figure 1. Our search identified 108 records. After removing 71 duplicate articles, 37 unique articles were screened by title and abstract. Of the 37 articles, 19 were excluded because they did not meet the eligibility criteria. The remaining 18 articles were reviewed in detail; 11 were excluded because they were review articles, four did not report on the relevant study group, and one was excluded due to unavailability of the full text. Finally, two studies were included in our meta-analysis.

Study Characteristics

Two eligible studies, both RCTs [10,11], were included in the meta-analysis. The baseline and critical characteristics of the included studies are shown in Table 1. Two large trials demonstrated the effectiveness of mavacamten in treating obstructive hypertrophic cardiomyopathy (OHCM). Olivotto I et al. performed a multicenter, phase 3 study enrolling 251 participants from 68 healthcare facilities in 13 countries. Mavacamten has demonstrated substantial improvements in exercise capacity, LVOT obstruction, NYHA functional class, and overall health status in individuals with OHCM [10]. The second study, conducted by Tian Z et al., included 81 volunteers and was a phase 3 trial conducted in China in 2023. Over 30 weeks, this trial demonstrated considerable improvement in the Valsalva LVOT gradient and other secondary efficacy targets. During each trial, mavacamten was administered orally via titration, beginning with a daily dose of 2.5 mg. Both groups responded well to mavacamten, and no additional safety concerns were identified in the Chinese cohort [11]. The results of these trials support the effectiveness and safety of mavacamten as a treatment for OHCM across a wide range of patient demographics.

**Table 1:** Characteristics of included RCTs. RCT: Randomized controlled trial; HOCM: Hypertrophic Obstructive Cardiomyopathy; NHCM: Non-Hypertrophic Cardiomyopathy; LVOT: Left Ventricular Outflow Tract; NYHA: New York Heart Association; LAVI: Left Atrial Volume Index; LVMI: Left Ventricular Mass Index; NT-proBNP - N-terminal Pro B-type Natriuretic Peptide.

Study ID	Study time and sites	Design and phase	Population					Dose, route, regimen	Duration of treatment	Conclusion
			Age	Condition OHCM, NHCM	Total No. of patients	Mavacamten	Placebo			
Olivotto I et al., (2020) [10]	From May 30, 2018, to July 12, 2019, in 68 clinical cardiovascular centers in 13 countries	Multicenter, double-masked, placebo-controlled, randomized, phase 3 study	More than 18	All patients in this study were OHCM	251	123	128	Dose titration scheme at weeks 8 and 14. Individualized doses of 2.5, 5, 10, or 15 mg were administered orally. Patients were evaluated every two or four weeks during the 30-week treatment period.	30 weeks	Mavacamten medication has substantially improved exercise capacity, LVOT obstruction, NYHA functional class, and overall health status in individuals with OHCM.
Tian Z, et al. (2023 [11]	Patients were enrolled between January 4 and August 5, 2022, conducted at 12 hospitals in China.	Phase 3, double-masked, randomized, placebo-controlled	Older than 18 years old	OHCM	81 patients	54 patients	27 patients	Orally once daily at a starting dose of 2.5 mg	30 weeks	Mavacamten significantly improved the Valsalva LVOT gradient vs. placebo for Chinese patients. All secondary efficacy endpoints were also enhanced. Mavacamten was well tolerated, with no new safety signals. This study supports the efficacy and safety of mavacamten

Risk of Bias Assessment

The quality of the two studies was evaluated using the Cochrane Risk of Bias Tool, the Jadad Scale, and the CONSORT Checklist [12-14]. According to all the instruments, both RCTs were of high quality and had a relatively low risk of bias (Table 2).

**Table 2:** Quality assessment of the studies. RCT: Randomized Controlled Trial; CONSORT: Consolidated Standards of Reporting Trials.

Quality Assessment Tool	Olivotto I et al. (2020) [10]	Tian Z et al. (2023) [11]
Cochrane Risk of Bias Tool		
Random sequence generation	Low risk	Low risk
Allocation concealment	Low risk	Low risk
Blinding of participants and personnel	Low risk	Low risk
Blinding of outcome assessment	Low risk	Low risk
Incomplete outcome data	Low risk (97.5% completed)	Low risk (97% completed)
Selective reporting	Low risk	Low risk
Jadad Scale		
Randomization	2/2	2/2
Blinding	2/2	2/2
Withdrawals and dropouts	1/1	1/1
Total Jadad Score	5/5	5/5
CONSORT Checklist		
Title and abstract	Clearly reported	Clearly reported
Introduction	Well described	Well described
Methods	Comprehensive	Comprehensive
Results	Clearly presented	Clearly presented
Discussion	Thorough	Thorough
Other information	Adequately provided	Adequately provided
Overall Quality Assessment	High-quality RCT with a low risk of bias	High-quality RCT with low risk

Evaluation of Efficacy Outcomes

**Figure 2:**
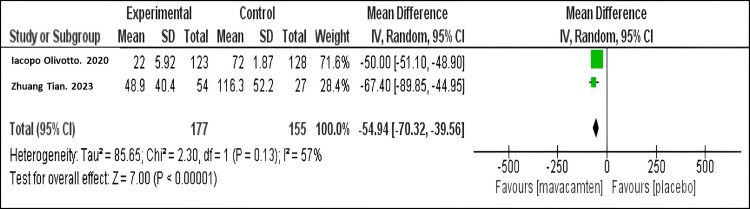
Forest plot of the mean change in the Valsalva LVOT gradient. Olivotto I et al. (2020) [10] Tian Z et al. (2023) [11] LVOT: Left Ventricular Outflow Tract.

Mean change in the Valsalva LVOT gradient: Our meta-analysis demonstrated that mavacamten therapy resulted in a marked decrease in the mean Valsalva LVOT gradient compared to placebo (mean difference MD = -54.94; 95% CI: -70.32, -39.56, P = 0.13), as shown in Figure 2. A similar finding was observed in both studies.

**Figure 3:**
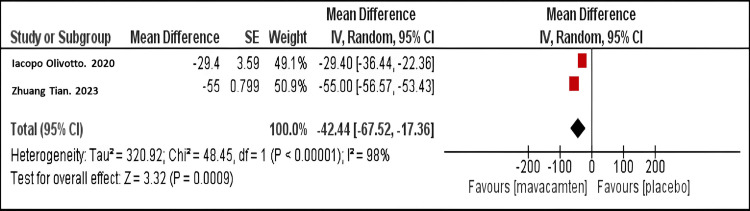
Forest plot of the mean difference in resting LVOT gradient. Olivotto I et al. (2020) [10] Tian Z et al. (2023) [11] LVOT: Left Ventricular Outflow Tract.

Mean difference in resting LVOT gradient: Our meta-analysis demonstrated that mavacamten therapy resulted in a marked decrease in the mean resting LVOT gradient compared to placebo (mean difference MD = -42.44; 95% CI: -67.52, -17.36, P<0.001), as shown in Figure 3. A similar finding was observed in both studies.

**Figure 4:**
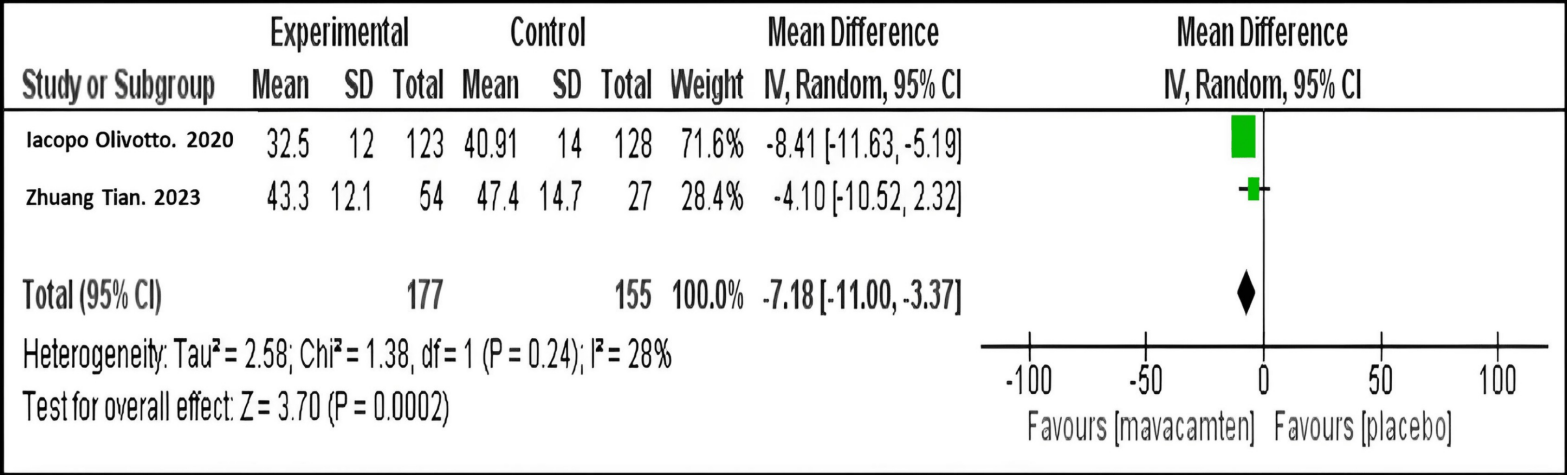
Forest plot for the mean change in LAVI. Olivotto I et al. (2020) [10] Tian Z et al. (2023) [11] LAVI: Left atrial volume index.

Mean change in LAVI: Our meta-analysis demonstrated that mavacamten therapy resulted in a significantly greater decrease compared to placebo (mean difference MD = -7.18; 95% CI: -11.00, -3.37, P = 0.24), as shown in Figure 4. The same finding was observed in both studies.

**Figure 5:**
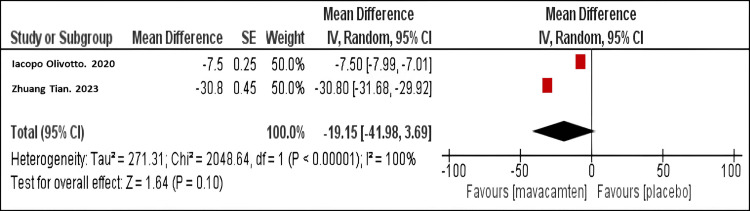
Forest plot of the mean difference in left ventricular mass index. Olivotto I et al. (2020) [10] Tian Z et al. (2023) [11]

Mean difference in the LVMI: Our meta-analysis demonstrated that mavacamten therapy resulted in a significantly more significant decrease in LAVI than placebo (mean difference MD = -19.15; 95% CI: -41.98, 3.69, P<0.001), as shown in Figure 5. The same finding was observed in both studies.

**Figure 6:**
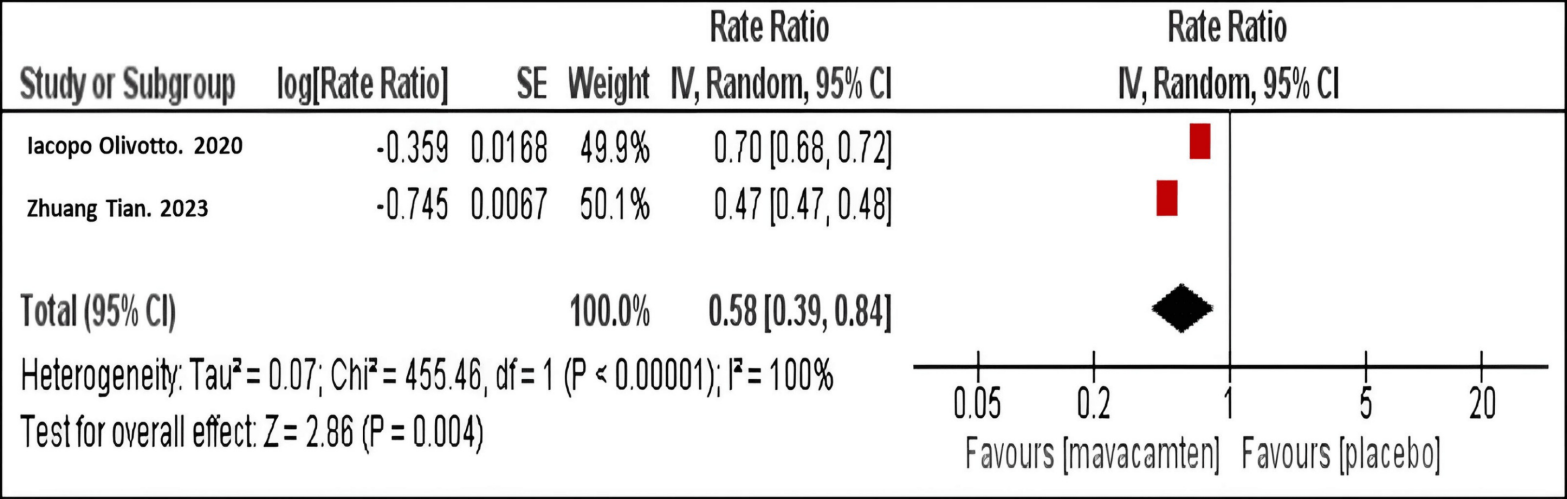
Forest plot for the mean ratio of N-terminal Pro B-type Natriuretic Peptide (NT-proBNP) levels. Olivotto I et al. (2020) [10] Tian Z et al. (2023) [11]

Geometric mean ratio of NT-proBNP level: Our meta-analysis demonstrated that mavacamten therapy significantly reduced NT-pro BNP levels compared to placebo (RR = 0.58; 95% CI: 0.39, 0.84, P< 0.001), as shown in Figure 6. The same finding was observed in both studies.

Safety outcomes: One study did not observe any adverse effects [10]. However, the authors mentioned that only two patients suffered from serious adverse effects due to mavacamten therapy, where one patient suffered from atrial fibrillation and the other from syncope. In another study, eight patients suffered from adverse effects due to mavacamten therapy [11], including atrial fibrillation (n = 2), atrial flutter (n = 1), sinus arrest (n = 1), sinus node dysfunction (n = 1), hypotension (n = 1), hemorrhoids (n = 1), and ankle fractures (n = 1).

Publication bias: The funnel plots of the pooled studies for various outcomes were symmetric, suggesting no obvious publication bias in reporting the outcomes of the included studies. Funnel plots for the efficacy outcomes are provided in the Appendix 1.

Discussion

The findings of this meta-analysis provide persuasive evidence that mavacamten is effective and safe in treating HOCM. As demonstrated by our findings, significant improvements were observed across various clinically essential endpoints, such as gradients in LVOT, cardiac structural characteristics, and biomarkers of cardiac stress.

Efficacy Outcomes

LVOT gradients: Our study showed that administering mavacamten significantly decreased the gradients of both the Valsalva maneuver and resting LVOT compared with placebo. The mean difference in the Valsalva LVOT gradient (MD = -54.94 mmHg; 95% CI: -70.32, -39.56) and the resting LVOT gradient (MD = -42.44 mmHg; 95% CI: -67.52, -17.36) are clinically significant and consistent with prior investigations. These findings align with those of the EXPLORER-HCM study, which demonstrated a decrease of 36 mmHg in the LVOT gradient after exercise [10]. The extent of the LVOT gradient decrease observed in our meta-analysis is more significant than that generally achieved with conventional pharmaceutical therapy alone, such as beta-blockers or disopyramide [15,16].

Cardiac structural parameters: Mavacamten showed beneficial effects on cardiac remodeling, as revealed by the considerable decreases in LAVI (mean difference = -7.18 mL/m²; 95% confidence interval: -11.00, -3.37) and LVMI (mean difference = -19.15 g/m²; 95% confidence interval: -41.98, 3.69). These structural changes are significant because they suggest that mavacamten may address the underlying pathophysiology of HOCM and relieve symptoms. The reduction in LAVI is particularly encouraging as it is associated with improved long-term outcomes in HOCM patients [17].

NT-proBNP levels: Additionally, the considerable reduction in NT-proBNP levels (relative risk = 0.58; 95% confidence interval: 0.39, 0.84) provides evidence of mavacamten’s favorable effects on cardiac stress and its potential impact on long-term prognosis. This finding is consistent with the EXPLORER-HCM study, which reported a 65% decrease in NT-proBNP levels with mavacamten therapy [10]. The degree of reduction in NT-proBNP observed in our study is more profound than what is typically seen with other HOCM medications, suggesting a stronger impact on cardiac stress [18].

Safety Profile

According to our findings, the safety profile of mavacamten appears to be favorable. In one trial, no adverse effects were observed [10], whereas in another study, a few serious adverse events were reported, including cardiac events such as atrial fibrillation, atrial flutter, and sinus arrest [11]. There is a minimal rate of significant adverse events, particularly considering the complex cardiac pathophysiology in HOCM patients. However, the fact that the included trials were only 30 weeks long necessitates the collection of long-term safety data to fully understand the risk profile of mavacamten.

Comparison With Existing Therapies

According to the findings of this meta-analysis, the effectiveness of mavacamten significantly surpasses that of conventional pharmaceutical treatments for HOCM. While beta-blockers and calcium channel blockers have been shown to be helpful in treating symptoms, their effects on LVOT gradients and cardiac remodeling are limited [19]. Disopyramide, often used in conjunction with beta-blockers, has been shown to significantly reduce LVOT gradients (mean reduction of 32 mmHg) [20], but these benefits do not match those observed with mavacamten in our study.

Furthermore, the structural improvements and decreases in biomarkers found with mavacamten suggest that disease modification is possible. This characteristic is often not associated with standard HOCM therapy. The unique mode of action of mavacamten as a cardiac myosin inhibitor directly addresses the hypercontractility characteristic of hypertrophic cardiomyopathy [21].

Clinical Implications

The findings of this meta-analysis have important implications for the management of HOCM. Compared to the various pharmaceutical treatments currently available, mavacamten appears to provide a more effective and potentially disease-modifying mechanism of action. Because of the significant decreases in LVOT gradients and heart structure improvements, mavacamten might delay or eliminate the need for more invasive treatments in some patients, such as septal myectomy or alcohol septal ablation [22]. Furthermore, the positive effects on cardiac remodeling and NT-proBNP levels suggest improved long-term outcomes, such as decreased risk of heart failure progression and arrhythmic events. This is especially pertinent considering the challenges associated with managing various clinical presentations of HOCM [23].

Limitations and Future Directions

Several limitations must be addressed, although our meta-analysis provides strong evidence for the short-term efficacy of mavacamten. First, the fact that our findings are based on only two RCTs, albeit large and well-designed, limits their generalizability. Second, the very short follow-up period of 30 weeks precludes any conclusions about the long-term efficacy and safety of the treatment. It is essential to conduct further research with a longer follow-up period to evaluate the drug's long-term effects and to monitor for potential late-onset adverse events. Additionally, the homogeneity of the study populations in terms of disease severity and ethnicity may limit the applicability of these findings to a broader range of HOCM populations. More studies are needed to determine whether mavacamten is effective in treating a wide variety of patient subgroups, including those with non-obstructive HCM and those at various stages of disease progression. Finally, comparative studies between mavacamten and existing invasive treatments (septal myectomy and alcohol septal ablation) are necessary to position mavacamten within the therapy strategy for HOCM.

## Conclusions

According to this meta-analysis, mavacamten is effective and safe for use in the treatment of HOCM, which provides significant support for these claims. Mavacamten is a promising novel medication with the potential to redefine the management paradigm for HOCM due to the remarkable improvements in LVOT gradients, cardiac structure, and indicators of cardiac stress. Although more extensive patient experiences and longer-term data are required, mavacamten offers substantial progress in the pharmaceutical therapy of this complex cardiac illness.

## Figures and Tables

**Figure 7:**
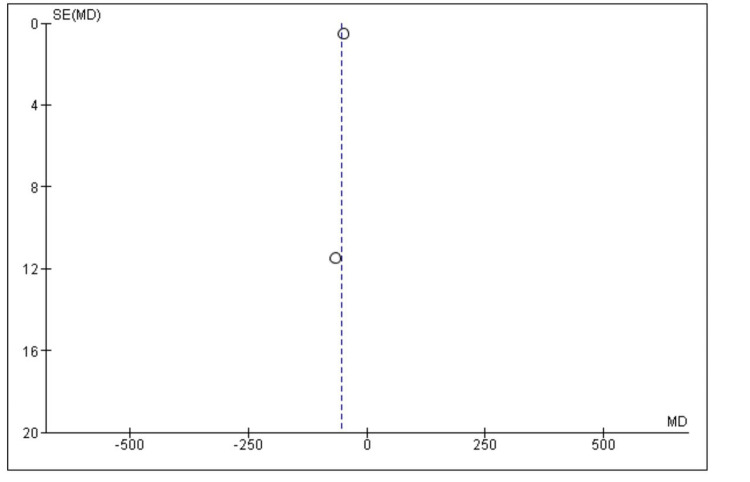
Funnel plot for the mean change in the Valsalva LVOT gradient. LVOT: Left Ventricular Outflow Tract.

**Figure 8:**
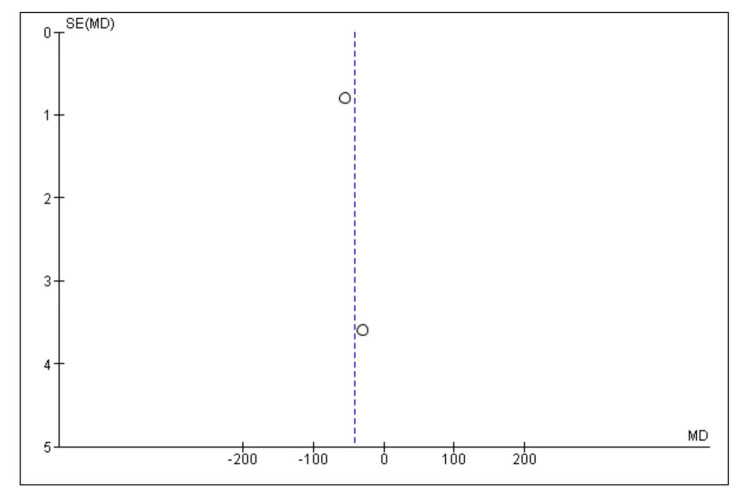
Funnel plot of the mean difference in resting LVOT gradient. LVOT: Left Ventricular Outflow Tract.

**Figure 9:**
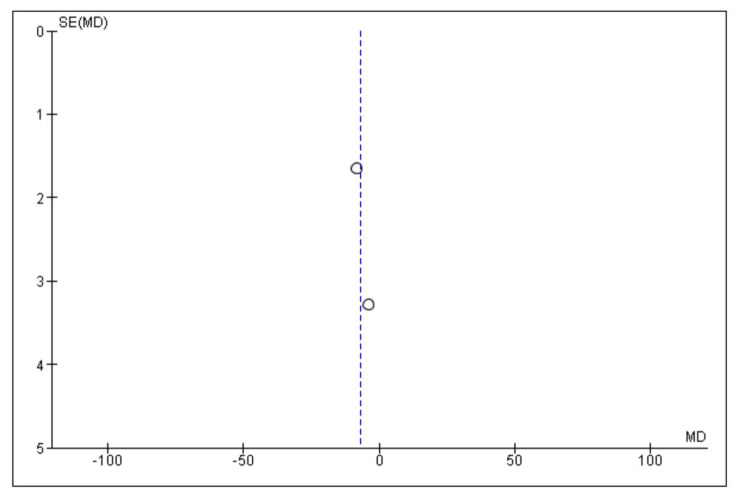
Funnel plot for the mean change in LAVI. LAVI: Left Atrial Volume Index.

**Figure 10:**
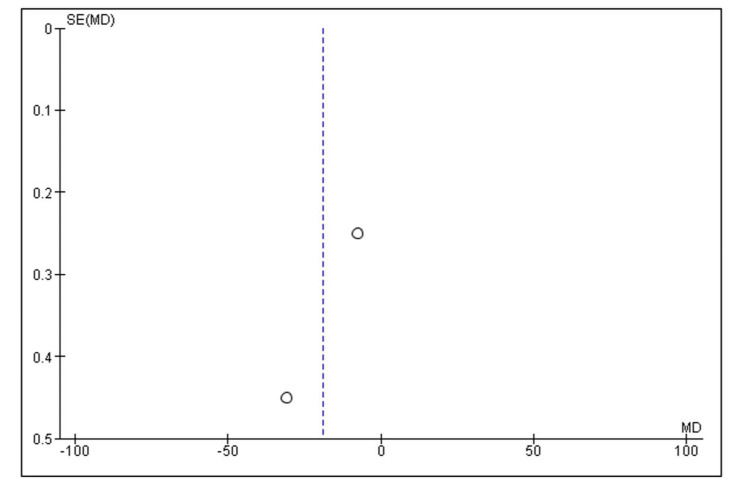
Funnel plot for the mean difference in the left ventricular mass index.

**Figure 11:**
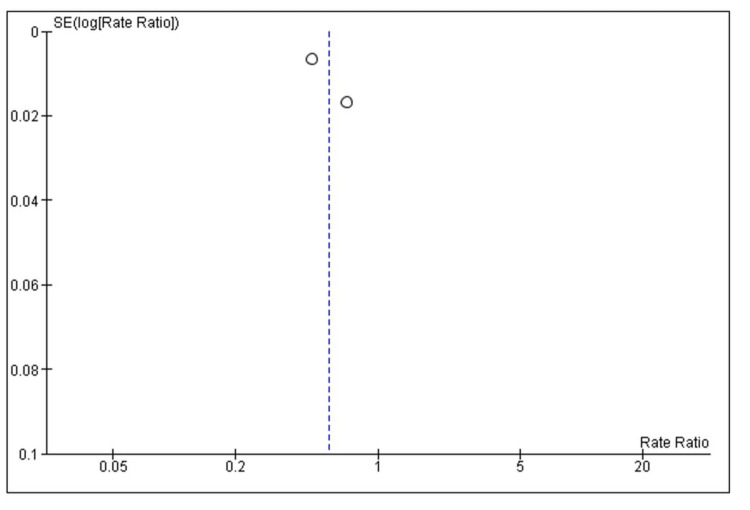
Funnel plot for the mean ratio of N-terminal Pro B-type Natriuretic Peptide (NT-proBNP) levels.

## Appendices

Appendix 1
