# Correction: Safety and Effectiveness of Mavacamten Use in Hypertrophic Obstructive Cardiomyopathy: A Systematic Review and Meta-Analysis

**DOI:** 10.7759/cureus.c321

**Published:** 2025-09-25

**Authors:** Tuqa Y Alharbi, Hadel A Alnadawi, Ghadah M Almutairi, Fatimah Y Altheyab, Osama H Aldoweesh, Omar S Alfehaid, Abdulmalik A Alhaj, Abdulaziz M Alotaibi, Ali M Al Zweihary

**Affiliations:** 1 College of Medicine, Qassim University, Qassim, SAU

This article has been corrected to the fix the following typos in the paragraph immediately preceding the PRISMA diagram:

- "...and one article could not be retrieved" removed as 0 articles could not be retrieved (as seen in PRISMA diagram). 
- "The remaining 17 articles were reviewed in detail; 11 were excluded because they were review articles, and four did not report on the relevant study group. Finally, two studies were included in our meta-analysis." has been corrected to the following: "The remaining 18 articles were reviewed in detail; 11 were excluded because they were review articles, four did not report on the relevant study group, and one was excluded due to unavailability of the full text. Finally, two studies were included in our meta-analysis."

